# A Case Series of Extrapulmonary *Mycobacterium* in Liver Transplant Recipients

**DOI:** 10.14309/crj.0000000000000571

**Published:** 2021-05-14

**Authors:** Kevin Lamm, Carl Jacobs, Mark W. Russo

**Affiliations:** 1 Division of Hepatology, Department of Medicine, Carolinas Medical Center, Charlotte, NC; 2 Department of Pathology, Carolinas Medical Center, Charlotte, NC

## Abstract

Liver transplant recipients are at increased risk of infection because of the immunosuppression required after transplantation. Infection by *Mycobacterium* species increases the morbidity and mortality of liver transplant recipients. The prompt recognition and diagnosis of opportunistic infection is necessary for good outcomes, particularly during periods of increased immunosuppression. The balance of immunosuppressive therapies during prolonged treatment with hepatotoxic medications has not been well studied and should be tailored for the unique clinical setting of each patient. The goal of treatment in these patients is to eradicate the disease and preserve allograft function.

## INTRODUCTION

*Mycobacterium* infection in liver transplant recipients has increased morbidity and mortality because of suppression of the cell-mediated immune system. Liver transplant recipients have an 18-fold increase in prevalence of tuberculosis (TB) and a 4-fold increased mortality because of the presence of immunosuppression.^1^ Atypical, non-TB *Mycobacterium* infection in liver transplant recipients has not been well studied. We present a case series of 3 liver transplant patients who received successful treatment for extrapulmonary *Mycobacterium* infection and retained allograft function despite suspending immunosuppression for up to 14 months.

## CASE REPORT

### Patient 1

A 30-year-old white man with a history of chronic biliary cholangitis secondary to right hepatectomy for hepatocellular carcinoma at age 18 presented 3 months after orthotopic liver transplant (OLT) with recurrent fevers, malaise, and poor oral intake. Laparotomy revealed significant inflammation and dense adhesions in the peritoneal cavity above the liver making it challenging to identify tissue planes. The pocket of infected material was above the liver in the midline and below the diaphragm. The area was drained, debrided, and cultures grew *Mycobacterium abscessus*. This bacterium does not respond to standard anti-TB medications. The susceptibilities showed resistance to all medications except for amikacin and tigecycline. There was intermediate susceptibility to cefoxitin and imipenem with a minimum inhibitory concentration dilution of 64 and 16, respectively.

Initial treatment included imipenem, amikacin, and tigecycline. Imipenem was discontinued indefinitely within a month of treatment because of drug-induced liver injury confirmed by biopsy. Amikacin was intermittently administered throughout the treatment course based on renal function. Cefoxitin was started once imipenem was discontinued and given a total of 6 months. Because of worsening fevers 6 months into therapy, tedizolid was initiated. Once tedizolid was initiated, fevers resolved. He was treated with a combination of cefoxitin, tedizolid, tigecycline, and amikacin for 18 months. Immunosuppression consisted of prednisone 5 mg alone for 14 months. Ultimately, the infection was treated, he retained allograft function, and he is doing well 4 years after liver transplant.

### Patient 2

**Figure 1.**
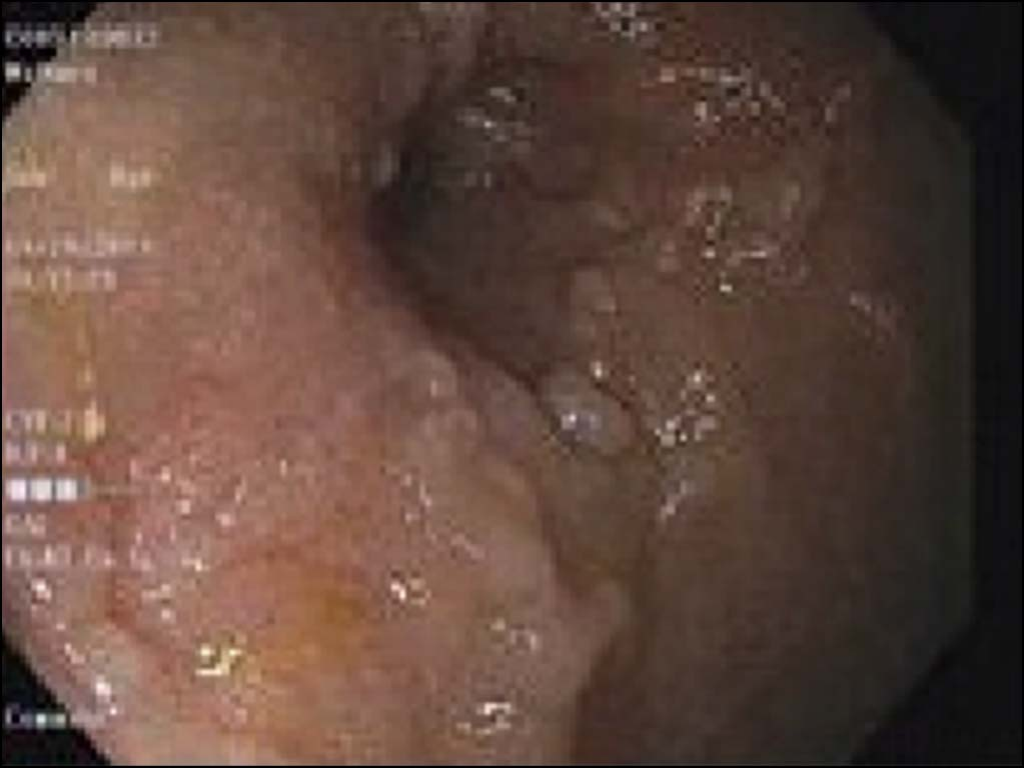
Esophagogastroduodenoscopy revealed a high-grade duodenal stricture.

**Figure 2.**
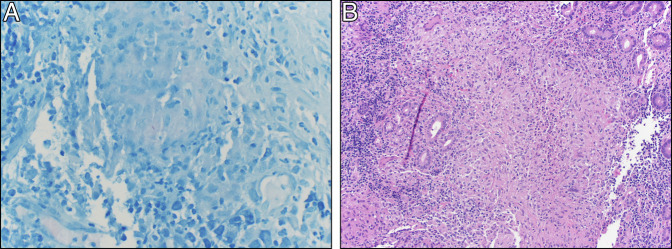
(A) Acid-fast *Bacillus* stain showing a single *Mycobacterium tuberculosis* organism and (B) hematoxylin and eosin stain showing severely active duodenitis ulceration and necrotizing and nonnecrotizing granulomatous inflammation.

A 52-year-old woman born in the Philippines with autoimmune hepatitis-primary biliary cholangitis overlap and 2 earlier episodes of acute cellular rejection presented 3 years after OLT with fevers and weakness. Computed tomography revealed marked thickening and mucosal enhancement of the small bowel with lymphadenopathy. Esophagogastroduodenoscopy revealed high-grade duodenal stricture (Figure 1). Biopsy showed focal granulomatous inflammation (Figure 2). Duodenal cultures returned positive for *Mycobacterium tuberculosis*. Immunosuppression consisted of prednisone 5 mg alone for 5 months. Her course was complicated by 30-pound weight loss that required 7 dilatations of the duodenal stricture and an episode of acute cellular rejection. She was successfully treated with 4-drug anti-TB therapy and has retained allograft function, now 9 years after liver transplant.

### Patient 3

**Figure 3.**
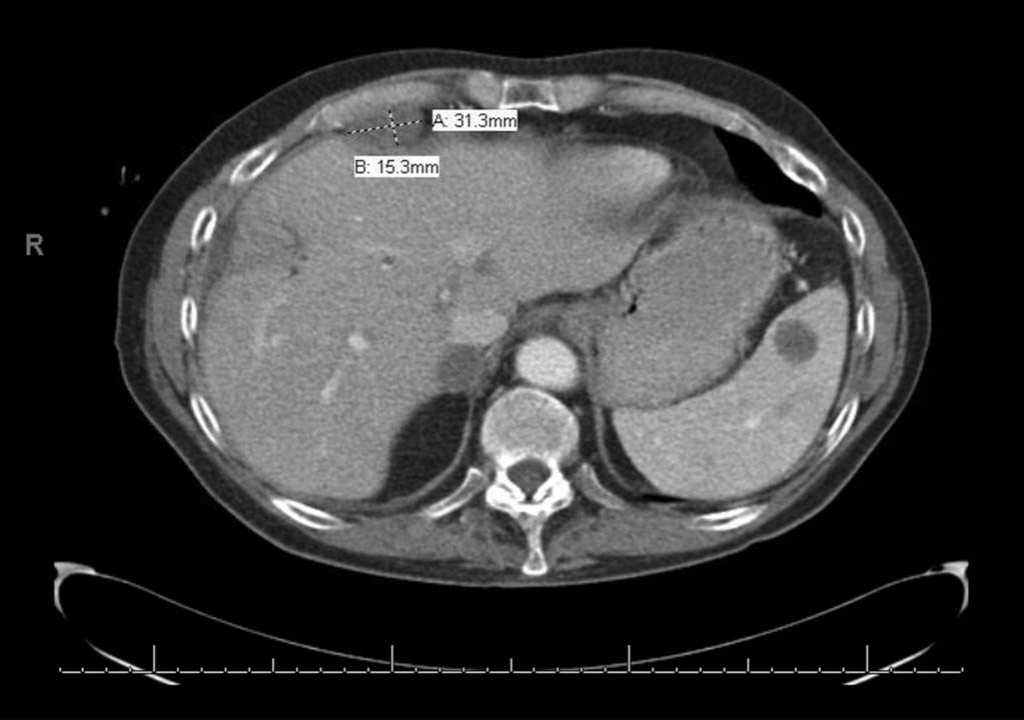
Computed tomography showing retroperitoneal adenopathy.

A 53-year-old white man with primary sclerosing cholangitis and ulcerative colitis presented 4 months after OLT with recurrent fevers. His course had been complicated by hepatic artery thrombosis, liver abscesses, and vancomycin-resistant enterococcus. Despite treatment of his known bacteremia and source control with percutaneous biliary drainage, he continued to have clinically significant fevers. Retroperitoneal adenopathy seen on computed tomography scan was evaluated by endoscopic ultrasound with fine-needle aspiration which confirmed granulomatous inflammation with necrosis (Figure 3). Acid-fast bacterial cultures returned positive for *M. tuberculosis*. Immunosuppression was withdrawn for 8 months although he remained on 5 mg prednisone daily. He completed a 78-week course of treatment for disseminated TB. He is doing well 9 years after liver transplant with retained allograft function.

## DISCUSSION

*Mycobacterium* species and treatment continue to be an essential problem in patients who have undergone liver transplantation. The short-term mortality rate for liver transplant recipients with active TB is estimated to be between 30% and 40%.^1,2^ Overall incidence ranges from 1% to 2.3%, whether in high-prevalence or low-prevalence areas.^2,3^ Approximately 85% of patients are diagnosed within 6 months, and many have a negative exposure history^4^; 34% of LT recipients diagnosed with TB had a previous episode of moderate-to-severe allograft rejection.^1^ The most common complications of treatment include drug hepatotoxicity and acute rejection.^1,3,5^

The etiology of *M. tuberculosis* in the above cases was likely reactivation because both patients were able to recall an exposure in their distant past. Although we serve in a nonendemic area, both patients had been tested before transplantation with QuantiFERON and were negative. The most common symptom of TB after liver transplant is persistent fever.^6^ TB can survive in lymph nodes and reactivate when the immune system is compromised. The most common abdominal manifestations include macroscopic ulceration, fibrosis, strictures, thickened intestinal walls, and lymphadenopathy.^7^

The management of TB infection in solid organ transplant recipients is complex because of the balance of the recipient's immune system and the extensive drug-drug interactions. Rifampicin and pyrazinamide have been shown to be the most effective drugs for standard TB therapy but are not ideal to use in liver transplant recipients because of significant drug interactions and intrinsic hepatotoxicity.^8^ Clinically significant hepatotoxicity is a well-described adverse effect of isoniazid, rifampin, and pyrazinamide. The combination of rifampicin/pyrazinamide is known to cause severe liver toxicity.^9–12^ Alternate antibiotics such as fluoroquinolones and tetracyclines have been used in cases of hepatotoxicity because of first-line agents.^8^

Acute rejection is attributed to drug-drug interaction with anti-TB therapy. Rifamycins interact with calcineurin inhibitors, mammalian target of rapamycin inhibitors, and corticosteroids by reducing serum levels of these medications, potentially precipitating graft rejection and dysfunction.^13–15^ In the meta-analysis by Holty et al,^1^ all cases of active TB were treated with ongoing immunosuppression of either cyclosporine or tacrolimus; 39% of cases required adjustment to the regimen in the setting of receiving rifampin.

The 3 cases described provide examples of the complex presentations of opportunistic infections and the difficult road to treatment while sustaining allograft function. Two of the 3 cases occurred within 4 months of transplantation; the third occurred after treatment of acute cellular rejection. When fevers and symptoms persisted despite antibiotic therapy, immunosuppression was withdrawn such that the regimen consisted of only low-dose prednisone for at least 8 months in all cases. Complete withdrawal of calcineurin inhibitors is a risk for allograft rejection and may not be suitable for all patients but should be considered when patients are not responding to antimicrobial therapy. There is an observational study that noted any reduction in the immunosuppressive therapy during bloodstream infection was associated with worse outcomes in liver transplant recipients.^16^ In contrast to bacterial infections, however, there is substantial evidence for the importance of T cells in the eradication of *Mycobacterium* infection.^17^ In the setting of overwhelming T-cell mediated diseases such as *Mycobacterium* infection or even cancer, we propose that withdrawing the calcineurin inhibitor can be considered when the recipient is not responding to medical therapy.

## DISCLOSURES

Author contributions: K. Lamm wrote the article and is the article guarantor. C. Jacobs reviewed the literature. MW Russo revised the article for intellectual content and approved the final article.

Financial disclosure: None to report.

Previous presentation: This case was presented at the American College of Gastroenterology Annual Scientific Meeting, October 13-18, 2017; Orlando, Florida and the Society of General Internal Medicine Southern Regional Meeting, February 22-24, 2018; New Orleans, Louisiana.

Informed consent was obtained for this case report.
